# Real-world implementation of a decision aid for treatment decisions in metastatic pancreatic cancer: a prospective multicenter study across expert centers

**DOI:** 10.1093/oncolo/oyag373

**Published:** 2026-09-29

**Authors:** Merlijn U J E Graus, Thijmen H T Broekman, Sietske van Nassau, Nicol Pepels-Aarts, Geert A Cirkel, Marcel den Dulk, Brigitte C M Haberkorn, Irene E G van Hellemond, Karlijn E P E Hermans, Marjolein Y V Homs, Maartje Los, Saskia A C Luelmo, Sasja F Mulder, Mirte M Streppel, Miriam L Wumkes, Johanna W Wilmink, Ignace H J T de Hingh, Liselot B J Valkenburg-van Iersel, Judith de Vos-Geelen, for the Dutch Pancreatic Cancer Group, M Besselink, B Groot Koerkamp, H van Laarhoven, J de Vos-Geelen, L Daamen, A Farina Saraqueta, C van Eijck, B Bonsing, M Homs, H Wilmink, S Luelmo, H van Santvoort, M Stommel, J van Hooft, V de Meijer, L Brosens, O Busch, I de Hingh, Q Molenaar, M Bruno, E Hendriksen, G Patijn, M Bijlsma, L van der Geest, G Kazemier, R Voermans, M Wumkes, E Manusama, M Streppel, L Brouwer-Hol, M den Dulk, I Verpalen

**Affiliations:** Division of Medical Oncology, Department of Internal Medicine, Maastricht University Medical Center, Maastricht, 6229 HX, The Netherlands; GROW—Research Institute for Oncology & Reproduction, Maastricht University, Maastricht, 6229 ER, The Netherlands; Department of Medical Oncology, Amsterdam UMC, Location University of Amsterdam, Amsterdam, 1081 HZ, The Netherlands; Department of Medical Oncology, University Medical Center Utrecht, Utrecht University, Utrecht, 3584 CX, The Netherlands; Division of Medical Oncology, Department of Internal Medicine, Maastricht University Medical Center, Maastricht, 6229 HX, The Netherlands; Department of Medical Oncology, University Medical Center Utrecht Cancer Center & St. Antonius Hospital Nieuwegein, Regional Academic Cancer Center Utrecht, Nieuwegein, 3430 EM, The Netherlands; Department of Surgery, School of Nutrition and Translational Research in Metabolism (NUTRIM), Maastricht University Medical Center, Maastricht University, Maastricht, 6229 HX, The Netherlands; Department of Medical Oncology, Maasstad Hospital, Rotterdam, 3079 DZ, The Netherlands; Department of Medical Oncology, Catharina Hospital Eindhoven, Eindhoven, 5623 EJ, The Netherlands; Division of Medical Oncology, Department of Internal Medicine, Maastricht University Medical Center, Maastricht, 6229 HX, The Netherlands; GROW—Research Institute for Oncology & Reproduction, Maastricht University, Maastricht, 6229 ER, The Netherlands; Department of Medical Oncology, Erasmus MC Cancer Institute, University Medical Center, Rotterdam, 3015 GD, The Netherlands; Department of Medical Oncology, University Medical Center Utrecht Cancer Center & St. Antonius Hospital Nieuwegein, Regional Academic Cancer Center Utrecht, Nieuwegein, 3430 EM, The Netherlands; Department of Medical Oncology, Leiden University Medical Center, Leiden, 2333 ZA, The Netherlands; Department of Medical Oncology, Radboud University Medical Center, Radboud Institute for Health Sciences, Nijmegen, 6525 GA, The Netherlands; Department of Medical Oncology, Amphia Hospital, Breda, 4818 CK, The Netherlands; Division of Medical Oncology, Department of Internal Medicine, Jeroen Bosch Hospital, Den Bosch, 5223 GZ, The Netherlands; Department of Medical Oncology, Amsterdam UMC, Location University of Amsterdam, Amsterdam, 1081 HZ, The Netherlands; GROW—Research Institute for Oncology & Reproduction, Maastricht University, Maastricht, 6229 ER, The Netherlands; Department of Surgery, Catharina Hospital Eindhoven, Eindhoven, 5623 EJ, The Netherlands; Division of Medical Oncology, Department of Internal Medicine, Maastricht University Medical Center, Maastricht, 6229 HX, The Netherlands; GROW—Research Institute for Oncology & Reproduction, Maastricht University, Maastricht, 6229 ER, The Netherlands; Division of Medical Oncology, Department of Internal Medicine, Maastricht University Medical Center, Maastricht, 6229 HX, The Netherlands; GROW—Research Institute for Oncology & Reproduction, Maastricht University, Maastricht, 6229 ER, The Netherlands

**Keywords:** pancreas adenocarcinoma, quality of life, decision aid, shared decision-making, treatment concordance

## Abstract

**Background:**

Treatment decisions in metastatic pancreatic adenocarcinoma (mPAC) involve complex tradeoffs between survival benefits and treatment burden. Shared decision-making (SDM) is crucial but challenging in emotionally charged and time-pressured consultations. This study evaluated the real-world implementation of a decision aid to support SDM in treatment decisions in mPAC care.

**Materials and methods:**

In this prospective, multicenter study across 10 Dutch expert centers, patients with mPAC and their clinicians used a newly-developed decision aid during consultations for treatment decisions. Data were collected on preferences, decision aid use, and final treatment choices, supplemented by surveys and interviews assessing feasibility and experiences. Outcomes included decision aid use, user experience, and concordance of final treatment decision with physician and patient preferences.

**Results:**

A total of 68 clinicians participated and 227 patients were counseled using the decision aid, of whom 181 patients consented to participate in the implementation study, and 135 patients used the online decision aid. Patients who received consideration time were more likely to use the decision aid (*P* < 0.01). Both patients and clinicians rated the decision aid positively (median score 8/10). Discordance between physician and patient preferences regarding treatment decisions was observed in 37%.

**Conclusion:**

A structured decision aid can be successfully implemented in real-world mPAC care and was highly valued by both patients and clinicians. Differences between clinician preference, patient preference, and ultimate treatment decision were frequently observed, highlighting the need to consider treatment decision-making as an ongoing process that allows time for reflection and dialogue.

Implications for practice This study shows that a structured decision aid for patients with metastatic pancreatic cancer is feasible and well accepted in routine clinical care, even within an emotionally demanding setting. Its use, particularly when combined with a structured reflection period, supports patients in clarifying their values and engaging in treatment decisions. Observed differences between patient and clinician preferences highlight the importance of follow-up consultations and ongoing dialogue. Effective implementation of shared decision-making in oncology therefore requires not only decision aids, but also sufficient time for reflection to align treatment decisions with what matters most to patients.

## Introduction

Metastatic pancreatic adenocarcinoma (mPAC) is characterized by a poor prognosis and limited palliative treatment options. Patients are often devastated when confronted with their diagnosis and associated short life expectancy. In parallel, a complex decision has to be made: whether to pursue intensive treatments that may provide survival benefit at the potential cost of significant toxicity and a time-invasive schedule, or to opt for best supportive care (BSC) with fewer therapeutic burdens but no cancer-directed action.^1–3^ These decisions are highly personal and complex, requiring thoughtful deliberation about acceptable tradeoffs while balancing survival, symptom relief, independence, and quality of life.

Yet, in pancreatic cancer care these decisions are made under conditions that leave limited room for reflection and mutual deliberation. Shortly after diagnosis, the initial consultation often coincides with tumor-related symptoms, making it an emotionally intense and cognitively challenging experience for patients. Studies show that patients forget up to 80% of the information provided, and of the content they do recall, only half is remembered accurately.^4^

Literature from mixed cancer populations consistently shows that oncology consultations often primarily focus on medical information, with less explicit attention to patients’ personal circumstances, values and preferences.^5^^,^^6^ In a Dutch survey among cancer patients, discussions about goals in life, fears, and supportive (non-cancer-directed) care options were reported to occur in about one-fourth of palliative treatment consultations.^3^^,^^5^^,^^6^ Clinical experience, however, suggests that, particularly in mPAC, supportive care and the option to forgo cancer-directed treatment may be discussed more routinely, possibly influenced by the prognosis and limited range of effective treatments. Whether and how patient values are systematically explored and integrated into decision-making in this specific setting is unknown.

Shared decision-making (SDM) provides a framework to support consultations involving complex and preference-sensitive decisions.^7^ By jointly weighing clinical evidence and personal values, SDM can reduce decisional conflict, improve patient satisfaction and compliance, and ensure that treatment decisions better align with patients’ individual goals.^8–10^ However, cancer care presents unique challenges for SDM, due to its emotional impact, clinical urgency, and uncertainty regarding both the effectiveness and the individual burden of available treatments.

Decision aids may help overcome some barriers in SDM by offering clear, accurate, and balanced information on available treatment options and by facilitating reflection on values and preferences.^11^ However, despite their demonstrated effectiveness, embedding decision aids into routine clinical care remains challenging, especially in high-pressure care trajectories, such as the initial consultation following a cancer diagnosis.^12–14^ Time constraints, emotional overload, and established clinical routines are often mentioned to hinder meaningful use.

Therefore, this prospective multicenter study aimed to evaluate the real-world implementation of a 3-part decision aid for patients with mPAC across 10 Dutch expert centers and to describe treatment preferences and decisions during its use. This may provide important insight into the integration of decision aids in routine oncology practice and inform strategies to support SDM in palliative cancer care.

## Materials and methods

### Study design and outcomes

This prospective, multicenter, mixed-methods study was conducted across 10 Dutch expert centers affiliated with the Dutch Pancreatic Cancer Group (DPCG). The study evaluated the real-world implementation of a decision aid for patients with mPAC in routine oncology practice. The primary outcome was concordance between physician preferences, patient preferences and the ultimate treatment decision. Secondary outcomes included associations between patient characteristics and clinician treatment preferences, as well as implementation across the participating centers and clinician- and patient-reported experiences with SDM and the decision aid.

### The decision aid

The *Deltaplan pancreatic cancer decision aid* (further, decision aid) was designed to support patients and clinicians during the treatment decision-making process in both first- and second-line systemic treatment consultations. The decision aid was developed through a structured, multi-phase process involving patients, clinicians, and advocacy groups and based on the Dutch guidelines for pancreatic cancer care. Full details are available in Appendix SI.

The decision aid consists of 3 components:

1. Summary handout: a concise 1-page printed overview of available treatment options, consisting of first-line palliative chemotherapeutic regimens FOLFIRINOX and Nab-Paclitaxel/Gemcitabine (NABGEM); BSC; second-line Nanoliposomal Irinotecan with 5-fluorouracil; and a reference to clinical trials, including their key benefits and risks. The medical oncologist indicated on the handout which treatment options the individual patient was eligible for. The handout was provided during the initial consultation with the medical oncologist or nurse specialist (Figure S1).
2. Online decision aid: an interactive web-based tool offering in-depth information about diagnosis, treatment options, real-world survival outcomes, side effects, treatment burden, and time investment.^15^ It also includes information on supportive care and pancreatic cancer-specific topics (eg, nutrition, biliary obstruction, pain management, and psychological support), alongside sections to help patients reflect on personal fitness, quality of life, personal values and peer experiences. Patients were encouraged to review the tool at home during a so-called “timeout period” to reflect on the information, discuss with relatives, and clarify preferences.
3. Follow-up consultation: a second consultation in which patient preferences and questions were discussed with their clinician and a shared decision was finalized based on informed deliberation.

### Implementation process

Ten DPCG-affiliated expert centers were selected to adopt the decision aid and participate in the study. Following medical-ethical approval (Medical Ethics Board azM/UM no. 2022-3546) and local institutional review board approvals, implementation process was initiated (Figure S2). At each site, intake meetings were conducted with the local principal investigator to discuss integration into existing workflows and role allocation, followed by structured kickoff meetings in which all involved clinicians received training on SDM principles, intended use of the decision aid, and embedding the 3-part workflow in their care pathway.

From March 28, 2023 to March 31, 2025, clinicians used the decision aid in routine care for all eligible patients. During this period, participating clinicians received monthly email updates with implementation statistics, practical tips, and patient feedback.

### Participants

Participating clinicians included all medical oncologists, oncology nurses, and nurse specialists involved in the care of patients with mPAC at each participating site. Eligible patients were adults (≥18 years) diagnosed with mPAC and facing a treatment decision (first- or second-line palliative therapy), and with sufficient command of the Dutch language to meaningfully participate in the study and complete study materials. All eligible patients received the decision aid (regardless of participation) and were asked to participate in the implementation study involving a 1-time patient survey, linkage of the survey to clinician-reported data from the consultation, linkage to online decision aid usage data, and follow-up data collection via electronic health records, including treatment choice and survival status.

### Data collection

Data were collected prospectively from both clinicians and patients. Full details on data collection are available in Appendix SI.

### Statistical analysis

Patient and clinician characteristics were presented as frequencies with percentages for categorical variables and medians with interquartile range (IQR) for continuous variables. Characteristics of those who used the online decision aid vs those who did not were compared using chi-square tests. Additional data on usage of and experience with the decision aid were reported descriptively. Clinician treatment preference, patient treatment preference (following their answers in the online decision aid; Figure S3) and ultimate treatment decision were summarized as frequencies and percentages. Discordance rates between clinician and patient treatment preferences were evaluated in patients who provided their treatment preference in the online decision aid. The number of discordant cases were reported, and the discordance rate was computed with 95% confidence intervals (CIs). Concordance and treatment differences between clinician and patiens were visualized using Sankey diagrams. Factors associated with clinicians’ treatment preference were explored using logistic regression models. Data was analyzed using IBM SPSS Statistics for Windows, version 28.0.0.0 (IBM CORP., Armong, NY USA) and R Studio (version 2023). *P* values of <.05 were considered statistically significant.

## Results

### Clinician characteristics

At start of the study 68 clinicians across 10 centers were involved in mPAC care: medical oncologists (69%), nurse specialists (19%), medical oncologists in training (6%) and oncology nurses (6%; Table S1). The majority worked in nonacademic hospitals (69%) and were female (78%). Of all clinicians, 40 (59%) completed the baseline survey. Respondents were mostly aged 36-45 (60%) with 6-10 years of oncology experience (35%). The majority reported having fewer than 5 new consultations with mPAC patients per month (83%) with a median consultation duration of 45 minutes (IQR 40-60). While 97% valued SDM, only 42% consistently applied it in practice.

### Patient inclusion and baseline characteristics

During the study period, 227 patients were offered the decision aid (Figure 1). These patients had a median age of 69 (IQR 61-75; Table 1) and 49% were female. Most had a WHO performance status of 0-1 (86%) and an expected life expectancy of >3 months (83%). Disease-related symptoms were present at first consultation in 72% of patients.

**Figure 1.**
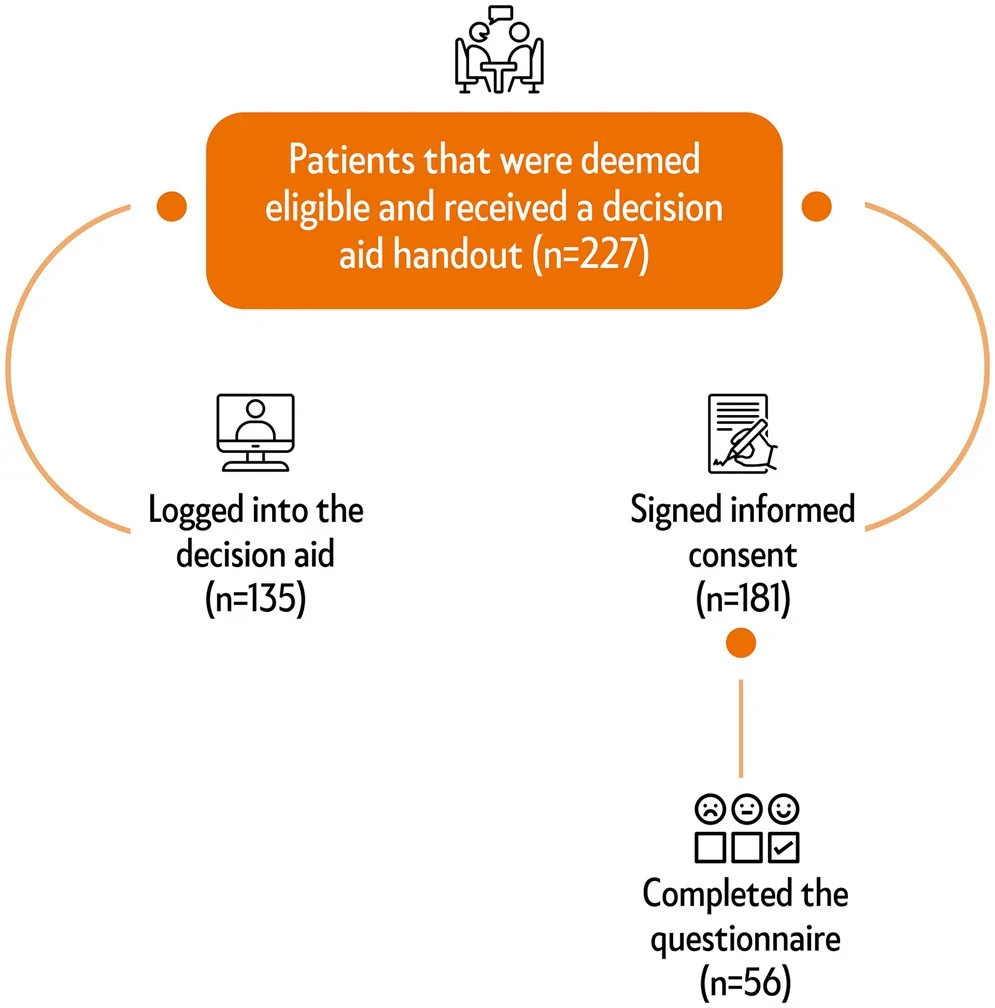
Study flow diagram on patients’ participation and inclusion.

**Table 1.** Baseline characteristics of all patients who received the decision aid for shared decision-making after metastatic pancreatic cancer diagnosis.

	All patients *n* = 227	Did not use online DA *n* = 92	Did use online DA *n* = 135	*P*-value
**Informed consent (*n*, %)**				
** Yes**	181 (80)	72 (78)	109 (81)	.77
**Age (median, IQR)** ^a^	69 (61-75)	66 (60-73)	68 (61-74)	.79
** <75 years (*n*, %)**	120 (53)	50 (54)	70 (52)	.82
** ≥70 years (*n*, %)**	107 (47)	42 (46)	65 (48)	
**Sex (*n*, %)**				
** Female**	111 (49)	44 (48)	67 (50)	.90
**WHO performance status (*n*, %)** ^a^				
** 0-1**	192 (86)	75 (82)	117 (88)	.33
** 2-4**	32 (14)	16 (18)	16 (12)	
** Missing**	*3* (*1*)			
**Life expectancy (*n*, %)** ^a^				
** ≤3 months**	37 (17)	15 (17)	22 (17)	1.0
** >3 months**	185 (83)	75 (83)	110 (83)	
** Missing**	*5* (*2*)			
**Disease symptoms (*n*, %)** ^a^				
** Yes**	160 (72)	63 (70)	97 (73)	.88
** No**	53 (24)	23 (26)	30 (23)	
** Unclear**	10 (5)	4 (4)	6 (5)	
** Missing**	*4* (*2*)			
**Hospital type (*n*, %)**				
** Academic hospital**	138 (61)	56 (61)	82 (61)	1.0
** Nonacademic hospital**	89 (39)	36 (39)	53 (39)	
**Available treatment options (*n*, %)** ^a^				
** FOLFIRINOX, NABGEM and best supportive care**	154 (68)	60 (66)	94 (70)	.59
** NABGEM and best supportive care**	69 (31)	29 (32)	40 (30)	
** Nal-iri/5FU and best supportive care**	3 (1)	2 (2)	1 (1)	
** Missing**	*1* (*0*)			
**Time-out before treatment decision (*n*, %)**				
** Yes**	169 (74)	56 (61)	113 (84)	**<.01**
** Time-out in days (median, IQR)**	4 (2-8)	4 (0-9)	4 (2-7)	.83
**Follow up consultation clinician (*n*, %)**				
** Medical oncologist**	132 (78)	48 (86)	84 (74)	.16
** Nurse specialist**	35 (21)	8 (14)	27 (24)	
** Oncology nurse**	2 (1)	0 (0)	2 (2)	

a Clinician reported at first consultation. Abbreviations: DA = decision aid; n = number; % = percentage; IQR = interquartile range; Nal-iri/5FU = nanoliposomal irinotecan / 5-fluorouracil.

Of the 227 patients, 181 (80%) consented to complete a 1-time survey and approve data linkage and 56 (31%) completed the survey (Table 2; Figure 1). Prior to discussing treatment options, 71% had synchronous mPAC and 71% had not received prior systemic therapy. Detailed sociodemographic characteristics are presented in Table 2.

**Table 2.** Baseline characteristics limited to patients who provided informed consent and submitted a survey (*n* = 56).

	*n* (%)
**Relationship status**	
** Married/relationship**	47 (84)
** Single/divorced/widowed**	9 (16)
**Living situation**	
** Alone**	12 (21)
** Together with partner, children and/or parents**	44 (79)
** In a care home (nursing home/assisted living facility/etc.)**	0 (0)
**Extra care**	
** Yes, professional home care**	5 (9)
** Yes, household help**	4 (7)
** Yes, help from relatives/neighbours**	5 (9)
** Yes, help from multiple sources**	5 (9)
** No extra help**	37 (66)
**Support network**	
** Yes, resident family**	14 (25)
** Yes, nonresident family/friends/neighbours (living within 20 km)**	5 (9)
** Yes, support from multiple sources**	30 (54)
** No support network**	7 (13)
**Level of education** ^a^	
** High**	21 (37)
** Intermediate**	20 (36)
** Low**	15 (27)
**Prior surgery for pancreatic cancer**	
** Yes**	16 (29)
** No**	39 (71)
**Prior systemic therapy for pancreatic cancer**	
** Yes, neo-adjuvant and/or adjuvant**	14 (26)
** Yes, palliative**	2 (4)
** No**	39 (71)
**Current disease-related symptoms**	
** Pain**	29 (53)
** Jaundice**	4 (7)
** Other**	4 (7)
** None**	18 (33)

a Level of education was based on definition of Statistics Netherlands (CBS) which is based on the International Standard Classification of Education.

b Due to rounding, percentages may not add up to 100.

Patients were treated in academic centers in 61% (Table 1). In 68% of cases the decision aid was provided for first-line treatment involving 3 options (FOLFIRINOX, NABGEM and BSC), 31% had 2 first-line options (NABGEM and BSC), and 1% received the decision aid for second-line treatment (Nal-iri/5FU and BSC). Most patients (74%) received a timeout before a treatment decision was made, with a median duration of 4 days (IQR 2-8).

### Decision aid use and patient feedback

Overall, 135 of 227 patients used the online decision aid (60%, Figure 1). They logged in for a median of 2 sessions (IQR 1-2), with 24% accessing it 3 or more times. The median time spent per session was 50 minutes (IQR 26-95), during which patients read a median of 85% of chapters (IQR 71-86%), and the majority (79%) completed the decision aid. Baseline characteristics were comparable between users and nonusers, except for the presence of a timeout before treatment decision (84% vs 61% respectively; *P* < 0.001; Table 1).

Most patients (90%) reported receiving the decision aid handout at the right time and 96% found it well-explained (Table S2). The majority considered the use of the handout pleasant in treatment discussions (90%) and helpful for providing structure and overview (84%). A minority found it overwhelming (10%) or confusing (4%). All patients who received a timeout, appreciated it.

After the first consultation, more than half of patients were interested to use the online decision aid (54%), whereas the others were neutral (29%) or not interested (17%). Among those who used the online decision aid, 59% did so with relatives and 38% alone. Nearly all patients (97%) found the decision aid easy to use, 85% required no assistance, and 88% found the amount of information appropriate. Most (91%) considered the information on anti-cancer treatment well-balanced between advantages and disadvantages.

Open-ended responses, provided by a minority of patients, indicated that the decision aid was mainly used to supplement the consultation, to review and reflect on information at home, and to gain a clearer overview of available options and what matters most in treatment decisions. It also supported discussions with relatives. Reasons for nonuse included expecting the tool to be too confronting or having already made a treatment choice.

Most patients reported that the decision aid made their treatment decision easier (94%; Table S2), and that it helped them in making a treatment decision (75%). Almost all patients (97%) agreed the decision aid should be provided to all patients in the future. The median overall rating from patients was 8/10 (IQR 8-9).

### Clinician perspectives

Among clinicians who completed the baseline survey, 87% believed the decision aid would positively impact SDM, while 13% expected no impact (Table S1). Three percent thought it would alter their prescribing patterns, while 54% disagreed and 43% were neutral. Similarly, only 3% believed patients would more frequently opt for a treatment different from the clinician’s preference. Most clinicians (95%) considered a timeout useful and feasible in daily practice. The majority (95%) felt the decision aid could be integrated into routine care and rated it a median of 8/10 (IQR 7-8).

### Treatment preferences and decisions

Overall, 37% (95% CI, 25%-51%; Table 3) of patients’ treatment preferences after using the online decision aid were different from the medical oncologist’s preference. Discordance rates varied substantially by treatment type: among patients who preferred FOLFIRINOX, 7% of oncologists had a different preference; among those preferring NABGEM, this was 50%; and among those preferring BSC, 90%. Among patients preferring BSC, concordance with clinicians was observed in only 1 case; in the remainder, clinicians preferred either FOLFIRINOX (50%) or NABGEM (40%).

**Table 3.** Discordance rate in first-line treatment preference between medical oncologist and patient after consultation and decision aid use for metastatic pancreatic adenocarcinoma diagnosis (FOLFIRINOX vs NABGEM vs best supportive care).

	Categories	Number of patients	Number discordant in preference of medical oncologist after first consultation	Discordance rate (95% CI)
**Treatment preference patients after decision aid usage**	Total	59	22	37% (25%-51%)
	FOLFIRINOX	27	2	7% (0%-19%)
	NABGEM	22	11	50% (32%-68%)
	Best supportive care	10	9	90% (70%-100%)

	Treatment preference of medical oncologists after first consultation (*n* = 59)					
Treatment preference of patients after decision aid usage	FOLFIRINOX chemotherapy (*n* = 40)		NABGEM chemotherapy (*n* = 17)		Best supportive care (*n* = 2)	
	*N*	Row%	*N*	Row%	*N*	Row%
**FOLFIRINOX chemotherapy (*n* = 27)**	25	93	2	7	0	0
**NABGEM chemotherapy (*n* = 22)**	10	46	11	50	1	5
**Best supportive care (*n* = 10)**	5	50	4	40	1	10

When comparing patient preferences with the ultimately chosen treatment, alignment remained strongest among FOLFIRINOX-preferring patients (91%), moderate for NABGEM (57%), and lowest for BSC (44%; Figure 2). Patients without a clear preference had mixed final treatment decisions (30% FOLFIRINOX, 40% NABGEM, and 30% BSC).

**Figure 2.**
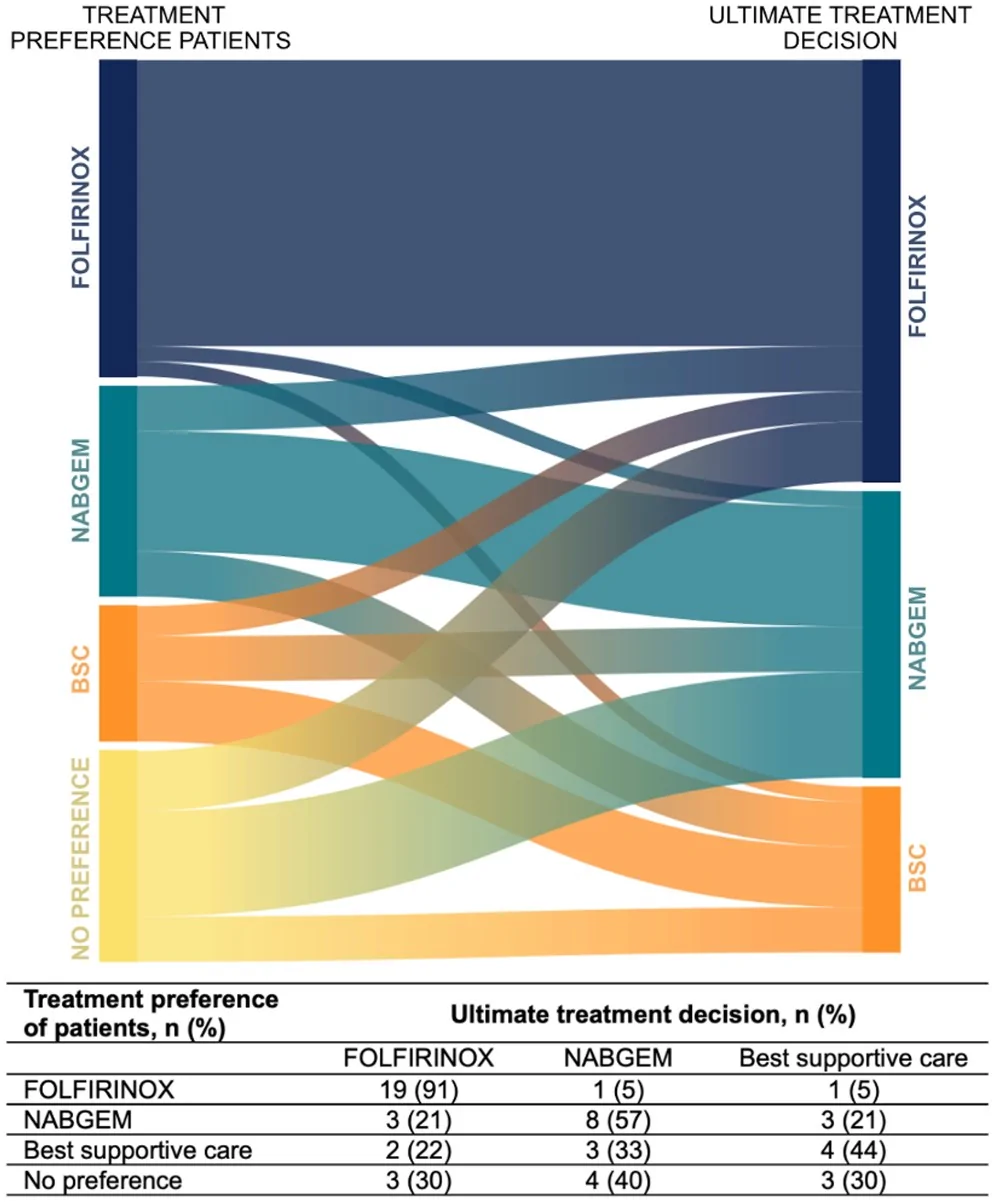
Discordance in treatment preference of patients vs ultimate treatment decision in first-line metastatic pancreatic adenocarcinoma (*n* = 58).

All patients for whom clinicians preferred BSC, received BSC (100%; Figure 3). Concordance was also high for chemotherapy preferences (75% for NABGEM and 65% for FOLFIRINOX). When clinicians expressed no preference, most patients (56%) ultimately received BSC.

**Figure 3.**
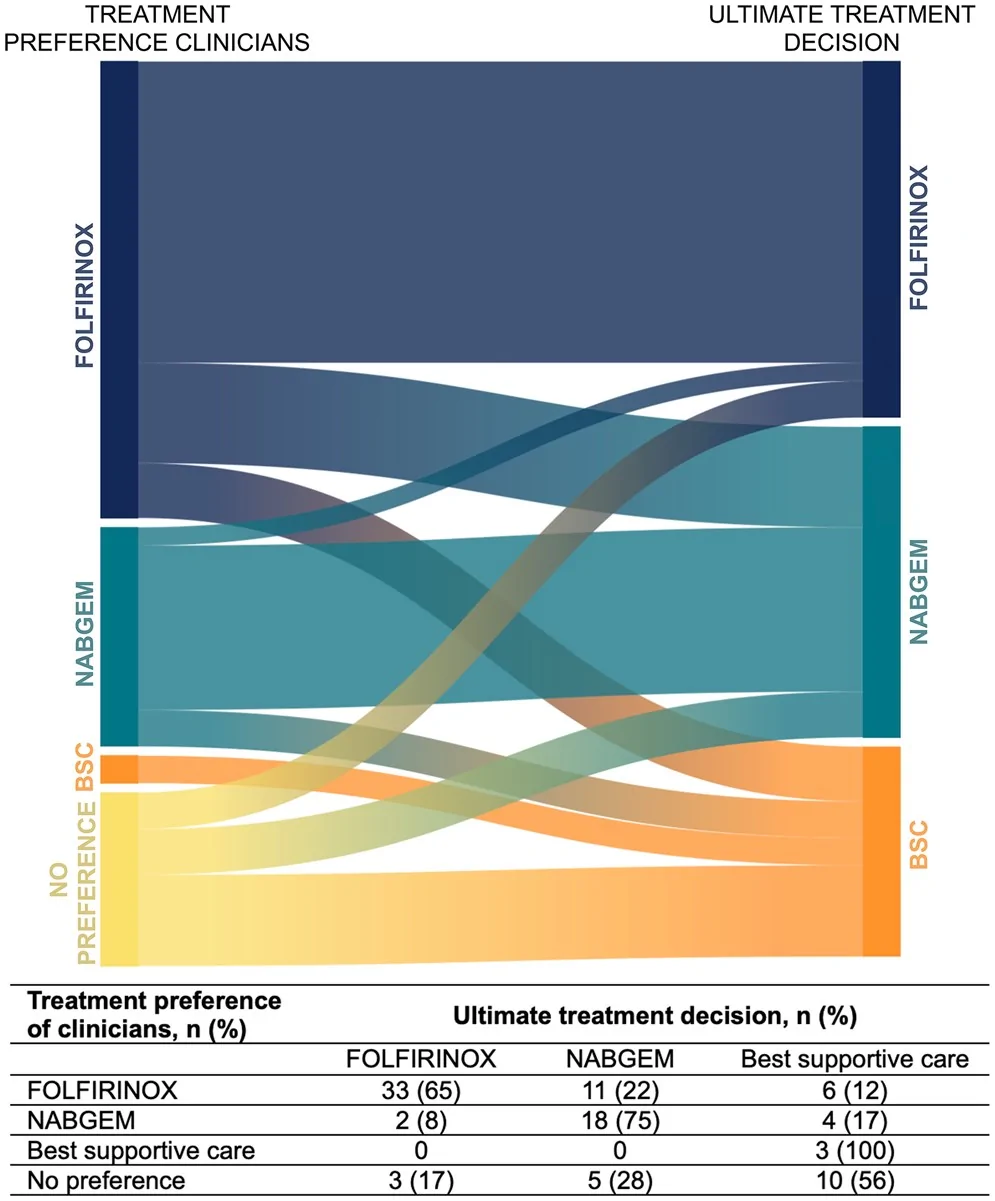
Discordance in treatment preference of clinicians vs ultimate treatment decision in first-line metastatic pancreatic adenocarcinoma (*n* = 97).

### Predictors of clinician preference

In univariable multinomial logistic regression, only estimated life expectancy was significantly associated with clinician treatment preference (Table 4). Clinicians were more likely to prefer BSC for patients with an estimated survival of < 3 months compared to those expected to live longer (OR, 11.6; 95% CI, 2.0-65.8; *P* = .01). Age, sex, WHO performance status, and disease-related symptoms showed no statistically significant association.

**Table 4.** Univariable multinomial logistic regression comparing predictors of Nab-Paclitaxel/Gemcitabine chemotherapy or best supportive care as preferred treatment of the medical oncologist vs FOLFIRINOX (*n* = 121).

		FOLFIRINOX (ref) vs Nab-Paclitaxel/Gemcitabine		FOLFIRINOX (ref) vs best supportive care	
Characteristics	*N*	OR (95%CI)^a^	*P* value	OR (95%CI)	*P* value
**Age**					
** <70 years**	75	0.51 (0.21-1.23)	.13	0.37 (0.08-1.70)	.20
** ≥70 years**	46	Ref.		Ref.	
**Sex**					
** Male**	66	0.75 (0.33-1.72)	.50	0.37 (0.09-1.60)	.18
** Female**	55	Ref.		Ref.	
**WHO performance status**					
** WHO 0**	37	0.54 (0.20-1.46)	.22	0.17 (0.02-1.80)	.14
** WHO 1-4**	84	Ref.		Ref.	
**Symptoms at initial diagnosis**					
** No**	27	0.92 (0.30-2.84)	.89	5.22 (0.89-30.42)	.07
** Yes**	94	Ref.		Ref.	
**Life expectancy**					
** ≤3 months**	16	1.66 (0.45-6.21)	.45	11.55 (2.03-65.81)	**.01**
** >3 months**	105	Ref.		Ref.	

Abbreviations: N, number; CI, confidence interval; OR, odds ratio; Ref, reference category.

a Odds ratios (OR) and 95% confidence intervals (CI) from a multinomial logistic regression model comparing Nab-Paclitaxel/Gemcitabine chemotherapy and best supportive care to FOLFIRINOX chemotherapy, which served as the reference category. The model included the following covariates: age, sex, WHO performance status, symptoms at initial diagnosis, and life expectancy. OR > 1 indicates increased odds of being in the respective category relative to best supportive care.

## Discussion

This prospective multicenter study evaluated the real-world implementation of a structured decision aid for patients with mPAC and described treatment preferences and decisions during its use across 10 Dutch expert centers. The decision aid was well accepted and highly valued by both patients and clinicians, even within emotionally charged setting of mPAC care. It also underscored the value of a structured timeout period between consultations, allowing patients to reflect on their values and preferences before making a final treatment decision. The finding that clinician preference and patients’ subsequent treatment preferences differed in approximately one-third of cases, illustrates that different perspectives may emerge during the decision-making process and warrant further exploration before a final treatment decision is reached.

The high uptake of the decision aid, with 80% of eligible patients providing consent and 60% actively using the online tool, demonstrates that structured SDM support is feasible, even in a population facing poor prognosis, high symptom burden, and limited treatment options. Most patients found the tool easy to use and often revisited it across multiple sessions, indicating good acceptability in this predominantly older and frail population. Importantly, baseline characteristics did not differ between users and nonusers of the online decision aid, suggesting that willingness and ability to engage with such tools are not restricted to specific patient subgroups. This is consistent with previous evidence showing that cancer patients, including those in palliative settings, value being involved in treatment decisions when supported by clear, balanced information, including the option of no cancer-directed treatment.^3^^,^^16–18^

Clinicians also rated the decision aid positively, acknowledging its usability and added structure to consultations. Although nearly all clinicians expressed positive attitudes toward SDM, only half reported applying it consistently in daily practice before implementation. This reflects the well-known gap between the intention and implementation of SDM.^12–14^ This gap often results from systemic barriers, such as time constraints, uncertainty about patient preferences, and the difficulty of end-of-life decisions.^3^^,^^19^ Furthermore, preexisting patient preferences, sometimes shaped by hope for exceptional outcomes or limited awareness of treatment-related burden, may challenge open deliberation during consultations. Decision aids may help bridge this gap by facilitating communication, reflection, and the explicit discussion of patient values, thereby supporting integration of SDM into daily care. This is particularly relevant in the Netherlands, where SDM is a national priority, as reflected in the 2025 vision of the Dutch Federation of Medical Specialists (Federatie Medisch Specialisten; FMS).^20^

A key finding of this study was the strong association between the use of a timeout period and actual decision aid engagement. Patients who received a dedicated reflection period between consultations were significantly more likely to use the online tool. This aligns with evidence from decision psychology showing that patients under emotional stress and time pressure often experience cognitive overload and are less able to deliberate about complex medical choices.^21–23^ Structuring care trajectories to allow patients to process information and discuss it with relatives may facilitate meaningful participation in decision-making.

Differences between physician and patient treatment preferences after reviewing the decision aid were observed in over one-third of cases, particularly among patients preferring BSC. This illustrates that patient and clinician perspectives may differ during the decision-making process. The first consultation for mPAC typically focuses on information provision and the awareness of treatment options, often occurring under emotional distress, leaving limited cognitive space for reflection. Following additional time for reflection and possible engagement with the decision aid, patients may express preferences that differ from the treatment advice recorded by clinicians after the initial consultation.

Without this structured process, there is a risk that treatment decisions rely predominantly on the clinical perspective established during the initial consultation, before patients have had the opportunity to fully consider and articulate their own preferences. Our findings show that clinician-reported treatment advice at this stage frequently differed from patients’ preferences reported later in the decision-making process. Notably, only 3% of clinicians expected that patients would more frequently opt for a treatment different from the clinician’s preference, whereas our study demonstrated this number is much higher.

A clear pattern emerged: clinician-reported treatment preference was largely concordant with the subsequent preferences of patients favoring systemic therapy, whereas concordance was substantially lower among patients who later expressed a preference for BSC. This pattern is consistent with previous literature showing that oncologists tend to overestimate patients’ willingness to pursue life-prolonging therapies and underestimate their preference for comfort-focused care or quality of life.^24^ For example, a study from the United States among patients with advanced neuroendocrine tumors showed that only a minority of patients prioritized survival over quality-of-life related factors as their main treatment goal.^25^ Moreover, almost half of these patients felt their treatment goal did not align with their physician’s. This may indicate that preferences for anticancer therapy are easier to recognize and operationalize, while preferences for no cancer-directed treatment and symptom-relief are more challenging to discuss and translate into practice. Possible explanations include prognostic uncertainty, therapeutic optimism, or an unconscious bias toward action among clinicians. However, this pattern should be interpreted in light of the study context, as patients with a clear indication for best supportive care alone were unlikely to be offered the decision aid, and clinicians may therefore have reported their preferred treatment within the subset of patients considered eligible for systemic therapy. Moreover, the current study cannot determine whether patients’ preferences changed during the reflection period, whether the decision aid contributed to such changes, or whether evolving clinical circumstances affected the feasibility of systemic treatment before the final decision was made.

The current study confirmed that clinicians’ initial treatment preferences were primarily driven by estimated survival rather than patient-centered factors such as age, WHO performance status, and disease-related symptoms. This highlights a tension between clinical prognostication and value-based care. While clinicians may base recommendations on minimal differences in expected survival, patients may prioritize quality of life, independence, or family time. A structured decision aid may help to bridge this gap by explicitly inviting patients to consider and communicate these priorities.^26^ Also, it remains uncertain how accurate physicians can judge early prognosis after a first consultation, and to what extent these estimates should dominate treatment recommendations.^27^

Differences between treatment preferences and ultimate decisions are not inherently indicative of (un)successful SDM. Rather, their occurrence highlights that decision-making should be regarded as an ongoing deliberative process rather than a single decision point. Follow-up consultations should not merely confirm a preference but provide opportunity to revisit treatment options, patient values, and clinical considerations before reaching a shared decision. Patient feedback supports this interpretation as most found that the decision aid made their treatment decision easier and improved communication with relatives and their clinicians. Importantly, it also complements the way the decision aid was designed; not as a tool that generates a single “correct” choice, but as a structured means to clarify values and preferences and to guide discussion with the treating physician. SDM is not synonymous with simply providing information as some patients might seek guidance rather than full autonomy. In contrary, it requires clinicians to balance autonomy with empathetic guidance as it prioritizes alignment of care with patient’s values and needs.^21^ These follow-up consultations for ongoing dialogue, combined with targeted clinician training on eliciting patient values, recognizing decisional conflict, and communicating uncertainty, may therefore support the translation of decision aids into truly shared decisions.^28^

This study is the first multicenter real-world evaluation of a decision aid in mPAC care, involving both patient- and clinician-reported data. Several limitations must be noted. First, the survey response rate among patients was modest, introducing a risk of selection bias as patients who were more willing or able to engage with the study may have been more likely to respond. The response rate should also be viewed in the context of the poor prognosis and high disease burden of this population. To minimize additional burden, subsequent follow-up was limited to one reminder. Second, the study lacked a control group limiting causal inference, but this design was chosen to avoid artificial comparisons, as patients generally report satisfaction with usual care when unaware of alternative approaches. Third, discordance was defined as a difference between the patient’s stated treatment preference and the medical oncologist’s reported advice. In the context of SDM, such differences do not necessarily reflect misalignment or suboptimal care, as final decisions may evolve through dialogue and clinical feasibility considerations. Moreover, oncologists were asked which treatment they advised, which may have been interpreted variably (for example, as their preferred option under the assumption that the patient wished to pursue active treatment). This potential ambiguity in interpretation may have influenced the observed discordance rates. Beyond advanced disease, future research should explore the applicability of such decision aids in earlier stages of pancreatic cancer, where treatment decisions may be equally complex.

This study shows that a structured decision aid is feasible and acceptable in real-world mPAC care, challenging assumptions that patients are too ill or emotionally burdened to engage in decision-making. The observed differences in patient-physician preferences and decisions emphasize the importance of structured decision moments, dedicated reflection time, and ongoing dialogue throughout the decision-making process. In addition, evaluating treatment regret and quality of life will be essential to determine the broader clinical impact of decision aids. Future studies should therefore incorporate validated patient-centered measures of decisional conflict, decision confidence, perceived involvement, and value concordance, as well as longer-term outcomes such as decision regret and quality of life, to more comprehensively evaluate decision quality and the clinical value of SDM interventions. In this way, decision aids have the potential to transform complex treatment conversations into deliberate, value-aligned decisions for patients facing pancreatic cancer.

## Supplementary Material

oyag373_Supplementary_Data

## Data Availability

The study protocol is available upon request to interested researchers via judith.de.vos@mumc.nl.
