# Supplementary material for: Real-world implementation of a decision aid for treatment decisions in metastatic pancreatic cancer: a prospective multicenter study across expert centers
Source: Oncologist. 2026 Sep 29;31(11):oyag373. doi: 10.1093/oncolo/oyag373 (PMC13652022; doi:10.1093/oncolo/oyag373)
Supplement: Supplementary file 1 [file oyag373_supplementary_data.zip › Supplementary_Material/DA_impact_Supplementary_Material_R.docx]

**Supplementary material**

Supplementary appendix I – Materials and Methods

Development of the decision aid

Data collection

Supplementary appendix II – Supplementary Tables
Supplementary table I Demographic characteristics of participating clinicians involved in
pancreatic cancer care

Supplementary table 2 Patients’ feedback on decision aid usage

Supplementary appendix III – Supplementary Figures

Supplementary figure 1 Summary handout of *Deltaplan Pancreatic cancer decision aid*

Supplementary figure 2 Implementation and study process per participating center

Supplementary figure 3 Method for indicating treatment preference for patients in the online	decision aid

**Supplementary appendix I – Materials and Methods**

**Development of the decision aid**

Prior to this study, the decision aid was carefully developed through a structured, multi-stakeholder process to ensure usability and acceptance. First, a needs assessment was conducted through in-depth interviews with patients who had previously faced treatment decisions for mPAC. These interviews explored their diagnostic and treatment trajectory, experiences with and perceived gaps in information provision, sources of supplementary information, and topics they would have preferred to avoid. In parallel, surveys were conducted among a national expert panel of multidisciplinary clinicians to map current practice, identify facilitators and barriers in decision making, and evaluate their perspectives on SDM and the potential added value of a decision aid.

Based on these findings, together with input from national guidelines and a project group including nationwide multidisciplinary professionals of the pancreatic cancer care network and representatives of the patient organization Living With Hope, the decision aid content was drafted. To enhance accessibility, the decision aid was reviewed by the Dutch ‘Easy Reading’ foundation, rewritten to B1-level language, and awarded the “Plain Language” certification.

Subsequently, the decision aid was tested with patients who had already undergone treatment decision making and with multidisciplinary clinicians. Again, in-depth interviews whilst participants used the online decision aid, provided structured feedback on clarity, completeness, and usability. A final round of surveys among the expert panel was conducted to validate content. After incorporating this feedback, the decision aid was finalized and ready for implementation.

**Data collection**

Data were collected prospectively from both clinicians and patients. Immediately after kick-off, clinicians completed a baseline survey (T0) via email, focusing on their view on SDM and decision aids, feasibility, acceptability, and perceived barriers and facilitators on implementing the current decision aid in routine care. Follow-up surveys at mid-implementation (T1, 5-6 months) and post-implementation (T2, ≥ 12 months) assessed changes in perceptions of SDM and experiences with the decision aid. All surveys were hosted on the electronic data capture tool Castor. Qualitative data were obtained during feedback meetings with healthcare teams at each site at T0, T1 and T2.

For patients, inclusion in the implementation study occurred at the first or second consultation in which treatment options were discussed. Approximately one week after consent, patients received a survey on sociodemographic and clinical background, self-assessed WHO performance status, experiences with the decision-making process, and feedback on the decision aid’s usability and helpfulness. A reminder was sent after one week if no response was received.

Clinicians also completed a brief anonymous survey after each eligible consultation, documenting baseline patient characteristics (age, sex, World Health Organization performance status (WHO PS), estimated life expectancy (<3 versus ≥3 months), presence of disease-related symptoms and eligible treatment options), and their preferred treatment. These surveys were completed regardless of patient consent, as no identifiable data were collected.

For patients who used the online decision aid, usage metrics (time spent, number of logins, sections read and stated treatment preference) were automatically recorded. Clinical follow-up data, including duration of timeout period, final treatment decision, survival, and reasons for early treatment discontinuation or switch (if applicable), were extracted from medical records. Data sources were linked using unique study identifiers.

**Supplementary appendix II – Supplementary Tables**

**Supplementary table 1** Demographic characteristics of participating clinicians involved in pancreatic cancer care (n=68)

|  | **n (%)** |
| --- | --- |
| **Profession** |  |
| Medical oncologist | 47 69) |
| Medical oncologist in training | 4 (6) |
| Nurse specialist | 13 (19) |
| Oncology nurse | 4 (6) |
| **Current working environment** |  |
| Academic hospital | 21 (31) |
| Non-academic hospital | 47 (69) |
| **Sex** |  |
| Male | 14 (21) |
| Female | 53 (78) |
| Other | 1 (2) |
| **Age** |  |
| ≤ 35 years | 7 (18) |
| 36-45 years | 24 (60) |
| 46-55 years | 5 (13) |
| > 55 years | 4 (10) |
| **Experience in oncology in years** |  |
| ≤ 5 years | 13 (33) |
| 6-10 years | 14 (35) |
| 11-20 years | 10 (25) |
| > 20 years | 3 (8) |
| **Number of monthly consultations with mPAC patients for discussing treatment options** |  |
| < 5 patients | 33 (85) |
| 5-10 patients | 6 (15) |
| > 10 patients | 0 |
| **Time for consultation with new mPAC patients in minutes** *Median (IQR)* | 45 (40-60) |
| **Do you use other decision aids in current clinical practice?** |  |
| Yes | 27 (70) |
| **Are you interested in shared decision making?** |  |
| Yes | 37 (97) |
| **Do you apply shared decision making in current clinical practice?** |  |
| Never | 0 |
| Sometimes | 1 (3) |
| Often | 21 (55) |
| Always | 16 (42) |
| **Do you think the decision aid will have an impact on shared decision making?** |  |
| No, no impact on shared decision making | 5 (13) |
| Yes, shared decision making will get worse | 0 |
| Yes, shared decision making will improve | 33 (87) |
| **Due to the decision aid, I expect to prescribe other treatment types more often than before** |  |
| Agree | 1 (3) |
| Neutral | 16 (43) |
| Disagree | 20 (54) |
| **Due to the decision aid, patients will more often choose a different type of treatment than the treatment of my preference** |  |
| Agree | 1 (3) |
| Neutral | 20 (54) |
| Disagree | 16 (43) |
| **Due to the decision aid, patients will more often choose FOLFIRINOX as first line treatment** |  |
| Agree | 1 (3) |
| Neutral | 20 (54) |
| Disagree | 16 (43) |
| **Due to the decision aid, patients will more often choose NABGEM as first line treatment** |  |
| Agree | 5 (14) |
| Neutral | 22 (60) |
| Disagree | 10 (27) |
| **Due to the decision aid, patients will more often choose best supportive care as first line treatment** |  |
| Agree | 2 (5) |
| Neutral | 27 (73) |
| Disagree | 8 (22) |
| **A timeout is useful if the patient’s medical situation allows it** |  |
| Agree | 35 (95) |
| Neutral | 2 (5) |
| Disagree | 0 |
| **The decision aid can be part of my normal routine** |  |
| Agree | 32 (87) |
| Neutral | 5 (14) |
| Disagree | 0 |
| **Rating of the decision aid so far** (score between 0-10) *Median (IQR)* | 8 (7-8) |

**Supplementary table 2** Patients’ feedback on decision aid usage

|  | n (%) |
| --- | --- |
| **Did you receive the handout (and therefore the decision aid) at the right time?** | 52 |
| Yes | 47 (90) |
| No, rather sooner | 5 (10) |
| No, rather later | 0 |
| **Was the handout explained to you in the consultation room?** | 52 |
| Yes, sufficiently | 50 (96) |
| Yes, but not sufficiently | 2 (4) |
| No, the handout was included but not explained | 0 |
| **How did you experience the information about treatment options provided through the handout?** | 50 |
| Pleasant | 45 (90) |
| Unpleasant | 5 (10) |
| **Experience with the handout** | 50 |
| It provided structure and overview | 42 (84) |
| It was overwhelming | 5 (10) |
| It caused confusion | 2 (4) |
| Other** | 1 (2) |
| **Experience with the timeout** | 53 |
| I had a timeout and appreciated it | 40 (76) |
| I had a timeout, but I would have preferred not to have one | 0 |
| I did not have a timeout, and I didn’t feel the need for one | 13 (25) |
| I did not have a timeout, but I would have preferred it | 0 |
| **At the end of the first consultation, was there an explanation given about how to use the online decision aid at home?** | 53 |
| My clinician gave me **the option** to log in, but **did not explain** the content | 13 (25) |
| My clinician gave me **the option** to log in, and **did explain** the content | 26 (49) |
| My clinician **instructed me** to log in | 1 (2) |
| My clinician **instructed me** to log in, and indicated we **would discuss it** at the next appointment | 6 (11) |
| My clinician **did not mention** the possibility to log in | 4 (8) |
| Other | 3 (6) |
| **Were you interested to log in to the decision aid after the first consultation?** | 52 |
| Yes | 28 (54) |
| Neutral | 15 (29) |
| No | 9 (17) |
| **I think it’s important to be involved in my treatment decision** | 53 |
| Yes | 49 (93) |
| Neutral | 4 (8) |
| No | 0 |
| **Did you log in to the online decision aid** | 52 |
| Yes | 37 (71) |
| No, I don’t know how to | 0 |
| No, but I will | 7 (14) |
| No, and I’m not going to | 8 (15) |
| **Logging in to the online decision aid was** | 34 |
| Easy | 33 (97) |
| Not easy but not difficult either | 1 (3) |
| Difficult | 0 |
| **Did you view the decision aid alone or with someone else?** | 34 |
| Alone | 13 (38) |
| With partner/(grand)children/relatives | 20 (59) |
| With my general practitioner | 0 |
| Other | 1 (3) |
| **Using the decision aid was** | 34 |
| Easy | 33 (97) |
| Not easy but not difficult either | 1 (3) |
| Difficult | 0 |
| **Did you need help from others to use the decision aid?** | 34 |
| Yes, a lot | 1 (3) |
| Yes, a little | 4 (12) |
| No, none | 29 (85) |
| **The amount of information in the decision aid was** | 32 |
| Too much | 3 (9) |
| Just right | 28 (88) |
| Too little | 1 (3) |
| **To what extent did the decision aid add anything to the information you received from your doctor?** | 32 |
| Nothing at all | 4 (13) |
| A little | 16 (50) |
| A lot | 11 (34) |
| Very much | 1 (3) |
| **To what extent did you have confidence in the accuracy of the information in the decision aid?** | 32 |
| Nothing at all | 0 |
| A little | 1 (3) |
| A lot | 24 (75) |
| Very much | 7 (22) |

| **To what extent was the information in the decision aid easy to read?** | 32 |
| --- | --- |
| Easy to read | 27 (84) |
| Neutral | 5 (16) |
| Difficult to read | 0 |
| **Were there words in the decision aid that you did not know and that were not made clear by the explanation?** | 32 |
| Yes, a lot | 0 |
| Yes, a few | 4 (13) |
| No | 28 (88) |
| **To what extent was the decision aid clear?** | 32 |
| Very clear | 5 (16) |
| Clear | 26 (81) |
| A little clear | 1 (3) |
| Not clear at all | 0 |
| **To what extent was the information on the treatment options balanced?** | 32 |
| The information focused too much on the disadvantages of the treatments (side effects). | 2 (6) |
| The information focused too much on the advantages of the treatment (life-extension) | 1 (3) |
| The information was well balanced between advantages and disadvantages | 29 (91) |
| **The decision aid made my treatment decision…** | 48 |
| Easier | 45 (94) |
| More difficult | 3 (6) |
| No influence | 0 |
| **Did the decision aid help you make a treatment decision** | 32 |
| Yes | 13 (41) |
| A little | 11 (34) |
| No | 8 (25) |
| **Was the decision aid of influence on your ultimate treatment decision?** | 32 |
| Yes | 9 (28) |
| A little | 12 (38) |
| No | 11 (34) |
| **Did your previous treatment preference change after using the online decision aid?** | 32 |
| Yes | 1 (3) |
| A little | 2 (6) |
| No | 26 (81) |
| I did not have a treatment preference yet | 3 (9) |
| **Did the decision aid make it easier to talk to friends and family about your diagnosis?** |  |
| Yes | 13 (41) |
| A little | 11 (34) |
| No | 8 (25) |
| **Did the decision aid make it easier to talk to your treating clinician?** | 32 |
| Yes | 12 (38) |
| A little | 12 (38) |
| No | 8 (25) |
| **The decision aid should be provided to all patients in the future** | 32 |
| Yes, to all patients | 31 (97) |
| To some patients | 1 (3) |
| No | 0 |
| **I would recommend the decision aid to other patients** | 48 |
| Yes, definitely | 37 (77) |
| Yes, probably | 9 (19) |
| No, probably not | 1 (2) |
| No, definitely not | 1 (2) |
| **Rating of the decision aid** (score between 0-10) |  |
| *Median (IQR)* | 8 (8-9) |

** Due to rounding, percentages may not add up to 100
** “I had already made my decision”*

**Supplementary appendix III – Supplementary Figures**

**Supplementary figure 1** Summary handout of *Deltaplan Pancreatic cancer decision aid*

**Supplementary figure 2** Implementation and study process per participating center

**Supplementary figure 3** Method for indicating treatment preference for patients in the online decision aid
